# Low steady-state oxidative stress inhibits adipogenesis by altering mitochondrial dynamics and decreasing cellular respiration

**DOI:** 10.1016/j.redox.2020.101507

**Published:** 2020-03-16

**Authors:** Raquel Fernando, Kristina Wardelmann, Stefanie Deubel, Richard Kehm, Tobias Jung, Marco Mariotti, Aphrodite Vasilaki, Vadim N. Gladyshev, André Kleinridders, Tilman Grune, José Pedro Castro

**Affiliations:** a Department of Molecular Toxicology, German Institute of Human Nutrition, Potsdam-Rehbrücke, 14558, Nuthetal, Germany; b German Center for Diabetes Research (DZD), 85764, München-Neuherberg, Germany; c German Center for Cardiovascular Research (DZHK), Partner Site Berlin, Germany; d Central Regulation of Metabolism Group, German Institute of Human Nutrition, Potsdam-Rehbrücke, 14558, Nuthetal, Germany; e Division of Genetics, Department of Medicine, Brigham and Women's Hospital and Harvard Medical School, Boston, MA, 02115, USA; f MRC-Arthritis Research UK Centre for Integrated Research Into Musculoskeletal Ageing (CIMA), Department of Musculoskeletal Biology, Institute of Ageing and Chronic Disease, University of Liverpool, UK

**Keywords:** Adipogenesis, Hyperoxia, Oxidative stress, Mitochondrial dysfunction, Rosiglitazone

## Abstract

Adipogenesis is a fundamental process of white adipose tissue function, supporting lipid storage and release, while avoiding its spillover and ectopic accumulation in tissues and organs. During aging adipogenesis is impaired and among other factors, oxidative stress contributes to this process. Adipogenesis requires functional and dynamic mitochondria; however, this organelle itself becomes dysfunctional during aging and accounts for most of reactive oxygen species (ROS) production. Here, we evaluated whether oxidative stress impairs adipogenesis through functional impairment of mitodynamics by utilizing hyperoxia as a continuous source of oxidative stress while maintaining cellular viability. This negatively impacted mitochondrial function, including respiration and dynamics and ultimately blocked adipogenesis. Interestingly, this state was reversible by using the antidiabetic drug, Rosiglitazone, which reduced oxidative stress, restored mitochondrial dynamics and respiration and augmented adipogenesis. Moreover, *in vitro* results were in agreement with *in vivo* models of oxidative stress and aging, in which mice depleted of the superoxide dismutase enzyme 1 (SOD1) and old wild-type C57BL/6JRj mice demonstrated the same trend of adipogenic potential. Importantly, in humans the results follow the same pattern, showing a downregulation of adipogenic markers during aging. Since the levels of oxidative stress and peripheral insulin resistance increase with age, while adipogenesis decreases during aging, our model helps to understand a possible way to overcome physiologically low, steady stress conditions and restore adipogenesis, avoiding accumulation of deleterious hypertrophic adipocytes in favor of beneficial hyperplasia.

## 1 Introduction

The white adipose tissue (WAT) is a key metabolic tissue, not only important for regulating energy storage, but also by providing energy for the organism upon demand. It is involved in regulating systemic metabolism by secreting adipocyte-related hormones such as adiponectin and leptin. Healthy adipose tissue relies on a continuous renewal of its cells, so-called adipocytes. In the WAT, adipocytes differentiate from multipotent mesenchymal stem cells (MSCs) in a multi-step process called adipocyte differentiation or adipogenesis. The view that continuous adipogenesis throughout life is detrimental is now outdated [1]. Adipogenesis is crucial for organism metabolic homeostasis and its diminishment represents a hallmark of obesity and insulin resistance [[2], [3], [4]].

Diminished adipogenesis can contribute to hypertrophy of mature adipocytes and eventually to free fatty acid release/spillover (lipotoxicity), resulting in insulin resistance across several tissues [5]. Furthermore, adipocyte hypertrophy is associated with decreased lifespan [6]. In mouse models of impaired adipogenesis, metabolic complications such as lipodystrophy and insulin resistance are frequent [7,8].

During aging, a progressive functional decline of WAT and the corresponding adipogenesis failure have been reported [1]. A likely mechanistic cause of these effects is the chronic, low-level state of oxidative stress (OS) observed during aging, which affects mitochondrial function and dynamics, contrary to acute, moderate oxidative stress, which activates insulin signaling in adipocytes and improves it function [9,10]. If this is true for preadipocytes, their capacity to differentiate into mature adipocytes would be impaired [11,12]. Unraveling how aging, through oxidative stress, affects adipogenesis is of utmost importance in order to avoid, delay or perhaps even treat metabolic complications such as obesity and insulin resistance. A possible cause for the gradual increase of chronic oxidative stress in adipose tissue is mitochondrial dysfunction. Generally, mitochondria are considered to be closely connected to cellular ROS production, due to their continuous action during metabolism and over time [13]. High ROS levels produced by dysfunctional mitochondria have been suggested as the main cause of aging [14], resulting from the accumulation of errors impinged on biomolecules. Mitochondria are also crucial for adipogenesis as they are the main source of energy for differentiation, a process which requires high levels of ATP [15,16]. Interestingly, the aging process and adipose tissue dysfunction share similar features; for example, obesity contributes to reduced lifespan, and the clinical outcomes closely resemble those usually found in aged organisms [17]. Therefore, obesity might result from adipose tissue dysfunction via functional impairment of mitochondria, ultimately causing excessive ROS production and leakage. Various studies have shown the advantages of using hyperoxia as a model of inducing oxidative stress (O_2_ levels higher than 21% in cell culture) [18]. Hyperoxia is known to dysregulate the mitochondrial respiratory chain through the reaction of oxygen with electrons that leak from the electron transport chain, for example from NADH or ubiquinone, which leads to the production of superoxide anion radicals [13,[18], [19], [20]]. Therefore, we used hyperoxic conditions as a chronic model to induce oxidative stress while maintaining cellular viability.

In this study, we aim to understand how OS affects mitochondrial function and adipogenesis. We found that under persistent non-toxic oxidative stress (8 days under hyperoxia), mitochondrial dynamics were impaired. This work is important by providing, for the first time, the evidence that OS can directly impair the WAT adipogenic process and that this might be an early mechanism of the development of metabolic alterations. We were able to show that the non-toxic, chronic OS could be overcome when treated with an antidiabetic drug, rosiglitazone. The PPARγ agonist rosiglitazone (R) ameliorated adipogenesis in cells under OS conditions by improving mitochondrial biogenesis, dynamics and respiration. In spite of being the major ROS source, mitochondria also display antioxidant defense mechanisms such as superoxide dismutase. It has been shown that mice lacking Cu/Zn-superoxide dismutase display increased levels of oxidative stress in several tissues and plasma [21], mostly related with higher levels of mitochondrial ROS, and decreased ATP levels and oxygen consumption [22]. Cu/Zn-superoxide dismutase-deficient mice also exhibit accelerated sarcopenia and decreased lifespan [23]. The early mortality of this mouse model seems to be associated with hepatocarcinogenesis in comparison to the wild type C57BL6 control mice [24]. Therefore, we took advantage of this oxidative stress mouse model and investigated whether the epididymal WAT (eWAT) of these animals had similar trends in expression of adipogenic and mitochondrial genes to our *in vitro* oxidative stress model. Taking advantage of the GTEx dataset, we could show that during human aging some of the main adipogenesis drivers are downregulated, both in men and women, in subcutaneous white adipose tissue, possibly reflecting a loss of adipogenic potential.

## 2 Material and methods

### 2.1 Experimental animals

Male C57BL/6JRj mice were purchased from Janvier Labs (France), at 16 and 105 weeks of age (8 and 7 mice, respectively) and kept in agreement with our laboratory animals guidelines for one week until sacrifice. Mice were euthanized with isoflurane followed by cardiac puncture. The eWAT was collected, frozen in liquid nitrogen and stored at -80 °C until processed. All experimental procedures were performed in accordance with the guidelines of German Law on the Protection of Animals and were approved by the local authorities (*Landesamt für Umwelt, Gesundheit und Verbraucherschutz*, Brandenburg, Germany).

Sod1 knockout (Sod1^−/−^) mice were originally generated at the University of Texas Health Science Center at San Antonio (UTHSCSA) and the Oklahoma Medical Research Foundation (OMRF). Mice were shipped to the Biomedical Services Unit, University of Liverpool. At both sites, mice were maintained under barrier conditions in microisolator cages on a 12 h dark/light cycle and were fed a CRM (P) rodent diet. Mice were euthanized by cervical dislocation, and eWAT was rapidly removed, frozen in liquid nitrogen, and stored at −80 °C. Procedures were performed in accordance with UK Home Office guidelines under the UK Animals (Scientific Procedures) Act 1986 and received ethical approval from the University of Liverpool Animal Welfare Ethical Review Body (AWERB).

### 2.2 3T3-L1 cell culture and differentiation

3T3-L1 preadipocytes were grown to confluency in DMEM (4.5 g/L glucose, 2 mM glutamine) (Thermo Fischer Scientific) supplemented with 10% FCS (Merck) (Normal Medium, NM). Five days later, adipogenesis was induced by incubation with DMEM containing 10% FCS, 170 nM bovine insulin (Sigma Aldrich), 1 μM dexamethasone (Sigma Aldrich) and 0.5 mM 3-Isobutyl-1-methylxanthine (IBMX, Sigma Aldrich) (Differentiation Medium, DM). After 2 days, the medium was replaced to NM supplemented with insulin for the remaining days (Maintaining Medium, MM). Cells were then allowed to differentiate for 6 more days in MM. For oxidative stress induction, cells were placed in hyperoxia (40% O_2_) or treated with antimycin A (Sigma Aldrich) for 5 days (20 nM) during DM and MM.

### 2.3 Oil Red O staining

To measure intracellular lipid content, cells were firstly washed in Phosphate Buffer Saline (PBS). Afterwards, they were fixed in formaldehyde 4% for 20 min. After fixation, cells were washed and incubated with Oil Red O dye (Sigma Aldrich) (3:2 isopropanol/H_2_O). To completely remove unspecific dye binding, cells were repeatedly washed in H_2_O for three times and allowed to air dry for 30 min. After drying, lipids were solubilized in DMSO (Sigma Aldrich) and the absorbance was read at 519 nm on a plate reader.

### 2.4 Measurement of ROS levels

Cells were incubated for 1 h in 3 μM H_2_DCFDA (diluted in NM). Afterwards, the media was carefully removed and cells were lysed in DMSO. Absorbance was read in the dark at 504/519 nm (emission/excitation. The obtained values were normalized to the cell number.

### 2.5 Isolation of RNA and real-time RT-PCR analysis from 3T3-L1 cells

Total RNA was extracted from 3T3 cells using the RNeasy Mini Kit (Qiagen, Hilden, Germany). From each sample, 1 μg of RNA was transcribed into cDNA with SuperScript II reverse transcriptase (Invitrogen) and poly (dT)15 primers. Expression of mRNA was analyzed by real-time RT-PCR using the Mx3005P system (Stratagene). PCR was performed with 10 ng cDNA, iQ SybrGreen Supermix (Biorad) and 0.5 μM of specific primers. Primers were designed using the Universal Probe-Library Assay Design Center (Roche) or from Harvard Primerbank. Mouse ribosomal protein, large, P0 (Rplp0) was used as internal normalization control. Primer sequences are referred to in Supplemental Table 1.

### 2.6 Isolation of RNA and real-time RT-PCR analysis from eWAT

mRNA was extracted from 30 mg of eWAT using Dynabeads™ mRNA purification kit (Thermo Fisher Scientific, Darmstat, Germany) according to manufacturer's guidelines. From each sample, 100 ng mRNA were transcribed with SensiFAST cDNA Synthesis kit (Bioline, London, UK). RT-PCR reactions were performed with Dream-Taq-Hot Start-DNA polymerase (Thermo Fisher Scientific, Darmstadt, Germany) and SYBR Green (Invitrogen, Carlsbad, USA). Ribosomal genes L13a, Rpl13a, and actin were used as internal normalization controls. The primer sequences used are the same as referred above.

### 2.7 Immunoblotting

Cells were lysed in lysis buffer, containing 0.1% Triton-100X, NaCl, EDTA and Tris pH 7.4 supplemented with phosphatase inhibitors. Protein concentration was determined using Bradford reagent. 10–15 μg of protein were separated by SDS-PAGE and transferred onto nitrocellulose membranes. For protein carbonylation assay, the protocol was followed according to Ref. [25]. Next, membranes were incubated with primary antibodies (Supplemental Table 2) overnight at 4 °C followed by incubation for 1 h at room temperature with fluorescent labeled secondary antibodies (Supplemental Table 2). Immunodetection was performed by Odyssey® **(**Li-Cor Biosciences).

### 2.8 Mitochondrial isolation

Cells were trypsinized (trypsin/EDTA) and centrifuged at 1200 rpm for 3 min, media was discarded and cells were resuspended 0.5 ml Fractionation Buffer (250 mM Sucrose, 10 mM Tris pH 7.5, 1 mM EDTA). Afterwards, cell were pottered 6x at 1000 rpm (on ice), centrifuged at 1000 g for 10min (4 °C) to remove unbroken cells. Supernatants were then centrifuged at 17000 g for 15 min (4 °C) (pellet = Mitochondria; supernatant = cytosolic fraction). Pellets were resuspended in 100 μl (Resuspension Buffer 250 mM Sucrose, 10 mM MOPS-KOH, 80 mM KCl, 5 mM MgCl_2_-Solution) and stored at -80 °C. Protein concentration was measured with Pierce 660 nm protein assay (ThermoFisher Scientific).

### 2.9 mtDNA content

DNA extraction from cells was performed using the Invisorb® Spin Tissue Mini Kit (Stratec) according to the protocol supplied by the manufacturer. DNA was amplified with specific primers using a SYBR green PCR Master Mix (Promega). The analysis was performed using ViiA 7 Real-Time PCR system (Applied Biosystems). Mitochondrial DNA content of mitochondrial-encoded genes, including NADH-ubiquinone oxidoreductase chain 1 (ND1), NADH-ubiquinone oxidoreductase 6 (ND6) and cytochrome c oxidase subunit 2 (COX2), were compared with a representative nuclear gene – Choline dehydrogenase (Chdh1). The primer sequences are found in Supplemental Table 1.

### 2.10 HPLC ATP/ADP levels

#### 2.10.1 Sample preparation

After counting, 1 million cells were used from each condition. Cellular pellets were resuspended in 500 μl H_2_O and boiled for 10 min and cooled on ice for 10 min. Next, the samples were centrifuged for 10 min at 12000 rpm. Afterwards, the supernatants were filtered (Waters Syringe Filters 0.2 μm) and 1 μl of each sample supernatant was determined by HPLC. This was conducted in a Agilent 1290 Infinity LC system under the following specifications; the column ZORBAX RRHD SB-Aq 2.1x50mm, 1.8 μM (Agilent) + UHPLC Guard 3 PK ZORBAX-SB-Aq 2.1x5 mm 1.8-μm (Agilent), solvent A (10 mM NH_4_H_2_PO_4_) and 2 mM PIC reagent A + 18% acetonitrile. For solvent B acetonitrile was used. The flow-rate was 0.2 ml/min, isocratic 99:1 and UV 254 nm. ATP and ADP concentration levels presented in μM were achieved after performing a calibration curve with standard ATP and ADP concentrations.

### 2.11 Mitochondrial dynamics

Cells were incubated in HBSS (Hank's Balanced Salt Solution with calcium and magnesium without phenol red, Gibco®) with 100 nM of Mitotracker Red (Thermofisher Scientific). After 30 min, cells were washed and placed in HBSS for mitochondrial dynamics visualization in a Zeiss confocal microscope *LSM780*, prepared with an atmosphere-chamber (temperature- and gas-control, 37 °C and 5% CO_2_, respectively). Analysis was done using *ZEN* standard software*.*

### 2.12 Seahorse assay

The Seahorse XF96 extracellular flux analyzer was used to monitor mitochondrial respiration, where oxygen consumption rate (OCR) was measured using Seahorse XF mito stress kit. This consisted of injecting sequentially the final concentrations of 2 μM oligomycin in order to inhibit ATP synthase, 1 μM carbonyl cyanide-4 (trifluoromethoxy) phenylhydrazone (FCCP) to uncouple oxygen consumption from ATP production and 0.5 μM Rotenone/Antimycin to inhibit complex I and III, respectively. The day prior to the assay, 7000 cells/well were seeded in a 96 well microplate in 100 μl medium. For background signal, four wells were left without cells. On the day of the experiment, the loaded sensor cartridge was calibrated and the cells were washed and replaced with Dulbecco's Modified Eagle's medium (Sigma-Aldrich) supplemented with 1 mM sodium pyruvate, 2 mM glutamine and 10 mM glucose. The cells were then incubated for 45 min at 37 °C in a non-CO_2_ incubator. After calibration, the microplate was placed into the seahorse analyzer. The Wave 2.4.0 software was used to analyze the data. Data was normalized to total protein amount and OCR was calculated by pmol/min/μg protein.

### 2.13 Antibody micro-array

3-4 millions cells (from T125 flasks), were trypsinized, washed and centrifuged. Then, 5 μl of protease inhibitor cocktail was added to each cellular pellet and stored at -80 °C. Pellets were then shipped to Sciomics for protein assessment. A volcano plot was used to display the differently expressed proteins from “control 8 days” and “oxidative stress 8 days” conditions. Data acquisition and analysis slide scanning was conducted using a Powerscanner (Tecan, Austria) with identical instrument laser power and adjusted PMT settings. Spot segmentation was performed with GenePix Pro 6.0 (Molecular Devices, Union City, CA, USA). Acquired raw data were analyzed using the linear models for microarray data (LIMMA) package of R-Bioconductor after uploading the median signal intensities. For normalization, a specialized invariant Lowess method was applied. Duplicate spots were averaged prior to the comparison of different experimental conditions. For analysis of the samples a one-factorial linear model was fitted with LIMMA resulting in a two-sided *t*-test or F-test based on moderated statistics.

### 2.14 Immunohistochemistry

eWAT was isolated, fixed in paraformaldehyde 4% and embedded in paraffin. Cross sections were cut using a microtome. The sections were first de-paraffinized by re-hydration in Roti-Histol (Carl Roth, Karlsruhe, Germany) and washed in decreasing serial dilutions of ethanol. For antigen retrieval, the slides were placed in citrate buffer (10 mM citrate acid and 0.05% Tween 20 in distilled water) at 95 °C for 20 min. Slides were cooled down for another 20 min at room temperature. Slides were incubated in a dark humified chamber for 1 h with blocking buffer containing 10% goat serum in antibody diluent (DAKO, Carpinteria, CA, USA). Slides were then incubated with anti-Pref-1/DLK1 primary antibody (R&D systems, Minneapolis, USA) overnight at 4 °C. Secondary antibody Alexa-fluor donkey anti-goat™ 546 IgG (Thermofisher Scientific) was then used for 1 h at room temperature. The sections were mounted with Roti-mount FluorCare DAPI (Carl Roth, Karlsruhe, Germany).

### 2.15 Statistical analysis

All figures and statistical analyses were performed using GraphPad Prism 6 software. Data represent in average three to four independent experiments (N = 3–4) and is presented as mean ± SEM. Statistical differences were considered when p ≤ 0.05 using the unpaired Student's t-test for the comparison within 2 groups or one-way ANOVA when more than 2 groups were compared.

### 2.16 GTEx analyses

We downloaded GTEx v7 from dbGap [26], and analyzed RNAseq quantifications in the adipose tissue, subdivided in two subtissues: subcutaneous (sWAT) and visceral (vWAT). We examined genes involved in adipogenesis, and tested the correlation between age and gene expression level separately for each subtissue and for female and males. P-values from Pearson's correlation tests were corrected by FDR considering the full list of genes examined. The R programming language was used for statistical tests and plots.

## 3 Results

### 3.1 (Pre)adipocytes under hyperoxia-induced oxidative stress do not show increased levels of adipogenesis

**Fig. 1:**
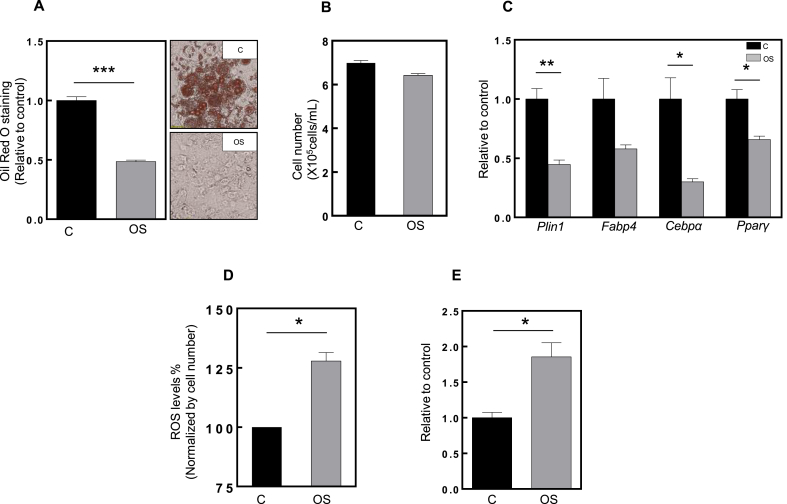
**Oxidative stress impairs adipogenesis in 3T3 cells.** The terms “C” and “OS” represent control (black column) and oxidative stress (grey column) and each condition is quantified relative to control. **(A)** The graphic depicts Oil Red O staining quantification of control and oxidative stressed cells, after 8 days differentiation. On the right, the upper panel represents the control (differentiated) cells and bellow the oxidative stress (non-differentiated) cells. The data from oxidative stressed cells were always normalized to the control (n = 9). **(B)** Graphic represents cell number of control and OS cells after 8 days differentiation. **(C)** mRNA expression of *Plin1, Fabp4, C/ebpα* and *Pparγ* under normoxia (C) and hyperoxia (OS) conditions. *Rplp0* was used as housekeeping gene. **(D)** ROS levels in C and OS cells. The values are expressed in percentage and each condition is normalized to cell number, and where OS is relative to C. **(E)** Densiometric quantification of carbonylated proteins (n = 3). Statistical significance was given as follows *p < 0.05, **p < 0.01, ***p < 0.001 (unpaired *t*-test) Values are presented as mean ± SEM.

Several studies have used hyperoxia as an oxidative stress inducer, mimicking chronic conditions during aging [27,28]. We established hyperoxic conditions (40% O_2_) for preadipocyte cultures as a continuous source of oxidative stress. These conditions suppressed 3T3-L1 cell differentiation after 8 days, as evidenced by Oil Red O staining (Fig. 1A), while avoiding loss of cell number (Fig. 1B). Mechanistically, hyperoxic conditions reduced the mRNA levels of adipogenesis transcription factors such as *Pparγ* and *Cebpα* as well as *Plin1*, an adipogenic marker (Fig. 1C).

### 3.2 Hyperoxia leads to increased oxidative stress

As expected, intracellular ROS levels were higher in oxidative stressed cells, and carbonylated proteins (a marker for oxidative damage) were also increased, confirming that our model indeed promotes oxidative stress (Fig. 1D and E, respectively). Moreover, by employing an antibody array, we found that many upregulated proteins in oxidative stress conditions are annotated with gene ontology (GO) terms related to response to stress and aging, reinforcing the idea that our hyperoxia model mimics oxidative stress (Supplemental Fig. 1). Remarkably, oxidative stressed cells could differentiate after a recovery period of 8 days in normal conditions (21% O_2_), suggesting that the model represents reversible oxidative stress. This was also consistent with the raise in lipid accumulation (Supplemental Figs. 2A) and a decline in carbonylated proteins (Supplemental Fig. 2B) after allowing the cells to recover in normal conditions for a further 8 days post stress. These results support the idea that metabolic dysfunction mediated by oxidative stress can be overcome under these conditions.

### 3.3 Oxidative stress does not affect gene expression in mitodynamics but is sufficient to induce mitochondrial dysfunction

**Fig. 2:**
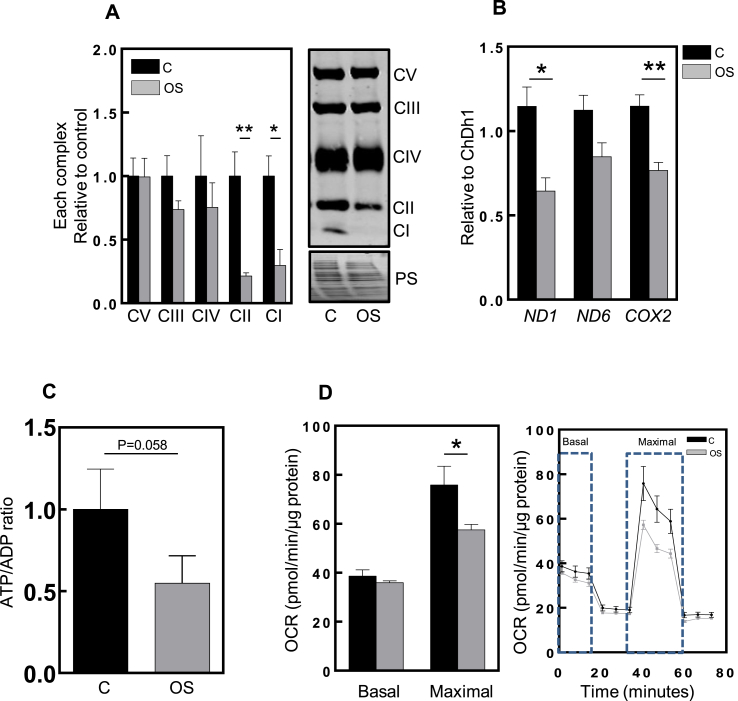
**Hyperoxia affects mitochondrial expression and function.** The terms “C” and “OS” represent the control (black column) and oxidative stress (grey column) and each condition is relative to the control. **(A)** Densiometric quantification of each electron transport chain complex in C and OS cells, relative to control. On the right, a representative western blot is observed as well as the Ponceau S staining, used as a loading control. **(B)** The graphic represents mitochondrial DNA content relative to a nuclear gene – ChDh1. **(C)** The graphic represents the ATP/ADP ratio measured by HPLC. The samples are normalized to the same cell number and OS is relative to C. Graphic bars result from three to four independent experiments. **(D)** Representative analysis of mitochondrial basal and maximal respiration of C and OS cells after 8 days differentiation. The graphic on the left represents the quantification of the oxygen consumption rate (OCR) of the basal and maximal respiration in pmol/min/μg of protein. The graphic on the right represents the schematic profile of mitochondrial respiration parameters over time (in minutes) of both control and oxidative stress (n = 14). Statistical significance was given as follows *p < 0.05, **p < 0.01, (unpaired *t*-test). Values presented as mean ± SEM.

Considering that mitochondria are important for both ROS production and adipogenesis, we decided to evaluate the effect of hyperoxia on mitochondrial function. Therefore, we characterized mitochondria according to levels of mitochondrial protein complexes; ATP levels; mitochondrial DNA copy as an indicator of mitochondrial mass; mitochondrial dynamics and biogenesis; and mitochondrial movement. In order to assess a possible shift between mitochondrial proteins, such as mitochondrial electron transport chain complexes, we first isolated mitochondria and performed Western blot analysis against five subunits of the electron transport chain. This analysis revealed reduced levels in some of the respiratory chain complexes when comparing oxidative stressed cells (non-differentiating) and controls (differentiating) cells (Fig. 2A). There is a clear downregulation of the complexes *I* and *II*. The reduction of these complexes and the loss in stoichiometry likely accounts for reduced respiration and mitochondrial dynamics [29,30]. Since mtDNA copy number is an indicator of mitochondrial mass [31], we determined mitochondrial content by analyzing the ratio of mitochondrial (*Nd1*, *Nd6*, *Cox2*) to genomic DNA content (*Chdh1*) (Fig. 2B). The results indicate a decline in *Nd1* and *Cox2* genes, suggesting a reduced mitochondrial content in cells exposed to hyperoxia. Due to the lower mitochondrial content and expression of ETC complexes, the ATP levels of control and OS-treated cells were investigated. The ATP/ADP ratio slightly decreased in OS treated cells yet did not reach statistical significance (Fig. 2C), which could explain the impairment in adipogenesis. We also tested whether oxidative stress is detrimental to mitochondrial dynamics and biogenesis. For this, we evaluated several proteins responsible for mitodynamics and genes such as mitofusin 2 (*Mfn2*), mitochondrial fission 1 (*Fis1*) and dynamin related protein 1 (*Drp1)*, as well as the peroxisome proliferator activated receptor gamma coactivator 1 alpha (*Pgc1α*), as a marker of mitochondrial biogenesis (Supplemental Figs. 3A–D, respectively). The levels of the outer mitochondrial membrane protein critical for mitochondrial fusion, Mfn2, were significantly lower in oxidative stressed cells. Moreover, the levels of mitochondrial fission protein Fis1, as well as the mitochondrial dynamin-related protein Drp1, were found to be lower, although not significantly, in oxidative stressed cells. This suggest an impairment of mitochondrial dynamics. The mitochondrial biogenesis transcription coactivator, PGC1α, also exhibited a tendency for lower protein levels in oxidative stressed cells (Supplemental Fig. 3D), corroborating the idea of reduced mitochondrial content. However, qPCR data for the respective mitochondrial dynamics and biogenesis genes did not show differences when comparing control and oxidative stress conditions (Supplemental Fig. 3F). This suggests that mitochondrial dysregulation is more likely to be caused by oxidative stress modifications rather than by altered transcriptional regulation, at least those related to mitochondrial dynamics. Mitochondria are active organelles that require mobility in order to carry out their cellular functions. Mitochondria are important for cell growth and differentiation, calcium signaling and regulation of the cell cycle [32,33]. Therefore, we set to assess whether oxidative stress affects mitochondrial mobility, employing a live cell mitochondrial-targeted dye. The stained mitochondria in cells were then acquired by imaging every 5 s, which was compiled in the form of a video. The results showed that oxidative stressed cells display an increase in mitochondrial rigidity (less movement), less fusion and more of a fission-like state compared with control cells (Supplemental Fig. 3E). Considering the evaluation of several mitochondrial parameters, such as mitochondrial function, content and dynamics, and the fact that mitochondria were affected by oxidative stress, we assessed another important factor, the mitochondrial respiration capacity. This was analyzed using a Seahorse XF96 assay. Sequential injections of different modulator compounds that target complexes of the electron transport chain were performed. The measurement started with the mitochondrial oxygen consumption rate (OCR) in resting conditions (basal respiration), in which differences were not observed between control or oxidative stressed cells. After injecting oligomycin, OCR decreased in both conditions. This suggests the amount of O_2_ consumed by the proton leak did not differ between control and oxidative stress conditions. Next, FCCP increased the OCR to its maximal respiratory capacity (maximal respiration), which was significantly lower in OS cells. Finally, a mixture of rotenone and antimycin A was injected, inhibiting C*I* and C*III* and concomitantly blocking mitochondrial respiration, which did not differ between conditions. In conclusion, basal respiration levels did not differ between control and stressed cells, however, maximal respiration capacity was significantly lower in the OS cells (Fig. 2D), which may account for the observed loss of adipogenic capacity. These data support the view that the ATP/ADP ratio is not significantly affected by oxidative stress in these cells, as ATP production is not altered (calculated from oligomycin treatment), however we did observe a decrease in the ATP/ADP ratio, when measured by HPLC, yet it did not reach statistical significance (p=0.058). One explanation for this disparity might be differences in ATP turnover by OS cells. In fact, these changes in ATP turnover rates have been documented in mouse lung cells subjected to hyperoxia [34]. Another possible explanation could account for the higher usage of ATP in OS cells to maintain viability. This would include processes such as DNA repair, stress protein synthesis or homeostasis of ions levels.

Taken together, these results show that oxidative stress resulting from hyperoxia strongly induces mitochondrial structural rearrangements, leading to increased ROS generation and contributing to the impediment of adipocyte differentiation.

### 3.4 Rosiglitazone significantly improves adipogenesis in oxidative stressed cells

**Fig. 3:**
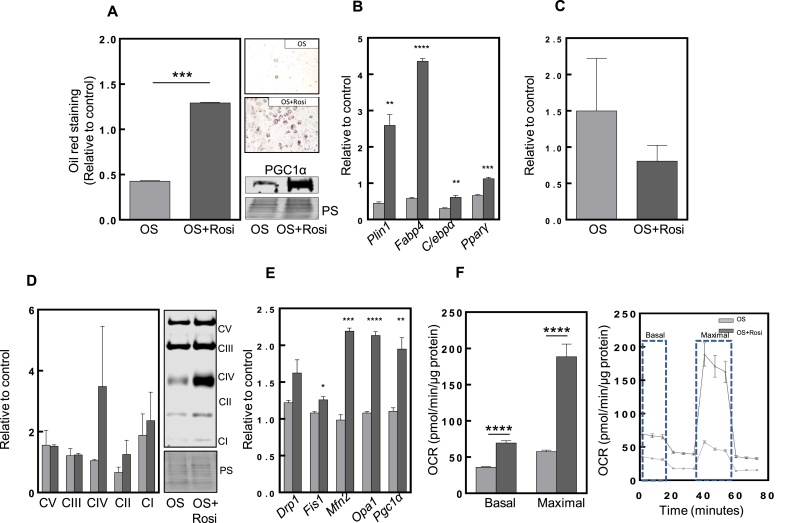
**Rosiglitazone rescues adipogenesis by increasing mitochondrial biogenesis, respiration and dynamics**. The terms “OS” and “Rosi” always represent the oxidative stress (grey column) and rosiglitazone (dark grey column), and each condition is relative to the control (despite not being shown). **(A)** The graphic represents the Oil Red O staining of OS cells and OS cells treated with Rosi. On the upper right is the picture of OS, and bellow is the picture of the cells treated with Rosi. Bellow the figures there is a representative western blot of PGC1α and the respective Ponceau S used as a loading control (n = 3). **(B)** The plot displays mRNA expression levels of *Plin1*, *Fabp4*, *C/ebpα* and *Pparγ* under hyperoxia and hyperoxia treated with Rosi. *Rplp0* served as housekeeping gene (n = 3). **(C)** The graphic represents densiometric quantification of carbonylated proteins. **(D)** Densiometric quantification of each electron transport chain complex relative to control. On the right, a representative western blot is observed as well as the Ponceau S staining, used as a loading control (n = 4). **(E)** The graphic represents mRNA expression levels of mitochondrial dynamics genes such as *Drp1*, *Fis1*, *Mfn2* and *Opa1* as well as mitochondrial biogenesis *Pgc1α* of OS cells and OS cells treated with Rosi. *Rplp0* served as housekeeping gene and the data is relative to the control (n = 3). **(F)** The graphic on the left represents the quantification of the oxygen consumption rate (OCR) of the basal and maximal respiration in pmol/min/μg of protein. The graphic on the right represents the schematic profile of mitochondrial respiration parameters over time (in minutes) of both OS and OS+Rosi (n = 14). Statistical significance was given as follows *p < 0.05, **p < 0.01, ***p < 0.001, ****p < 0.0001 (unpaired *t*-test). Values presented as mean ± SEM.

As mentioned before, *Pparγ* is a master regulator of adipogenesis. Since we verified its reduction under stress conditions, we decided to boost its activity by subjecting 3T3-L1 cells to the common anti-diabetic drug and agonist, rosiglitazone (R) [35]. We verified a significant increase in lipid droplet formation compared to non-R treated hyperoxic cells (Fig. 3A). Remarkably, there was an increase of the mitobiogenesis transcriptional coactivator marker, PGC1α, at protein level (Fig. 3A), as well as an increase fusion (Mfn2) and fission (DRP1) proteins (Supplemental Figs. 4A and B, respectively), suggesting an improvement of mitochondrial content and dynamics. Moreover, we observed by live-cell imaging that mitochondria were morphologically different in cells subjected to oxidative stress (Supplemental Fig. 3E-video available in online version). It could be clearly noted that the movement of stressed cells treated with R was restored, and the fission-like state now shifted towards a more fusion-like state, indicating an improvement of mitodynamics [36]. Interestingly, this corresponded to an increased mRNA expression of adipogenic markers (Fig. 3B) and a trend for reduced protein carbonylation, indicating a possible reduction in oxidative stress or to increased turnover of carbonylated proteins (Fig. 3C). The mitochondrial electron transport chain proteins remained mostly unchanged, but with an increased but non-significant response to R treatment for complexes *I*, *II* and *IV* (Fig. 3D). Furthermore, mitochondrial biogenesis (*Pgc1α*) and dynamic-related genes (*Fis1*, *Mfn2* and *Opa1*) were elevated (Fig. 3E). Interestingly, not only the maximal but also the basal respiratory capacity was significantly increased (Fig. 3F), suggesting a recuperation of the oxidative phosphorylation machinery and its function.

### 3.5 Impairment of adipogenesis is a result of mitochondrial malfunction

**Fig. 4:**
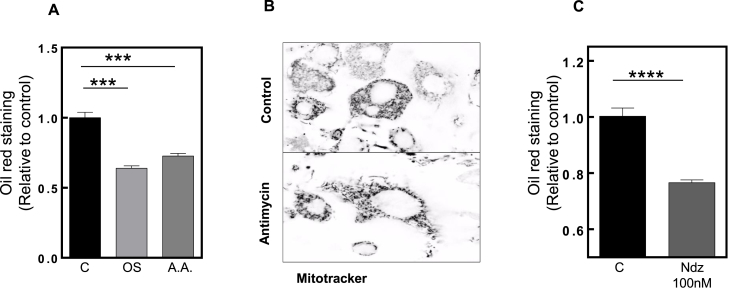
**Mitochondrial inhibitors impair adipogenesis.** **(A)** The graphic represents the comparison between C (black), OS (light grey) and 5 days treatment of 20 nM Antimycin A (A.A.) (dark grey). Each condition is relative to control. **(B)** The upper panel shows a representative video of mitochondria under 8 days normoxia conditions and the lower panel shows mitochondria under 8 days normoxia conditions treated with A.A. for 5 days. These videos can be seen in the online version (mitochondria stained with mitotracker). **(C)** The graphic represents Oil Red O staining of cells under normoxia conditions (black column) and cells under normoxia conditions treated with 100 nM nocodazole (Ndz) for 2 days (n = 3). Statistical significance was given as follows ***p < 0.001 (One-Way ANOVA) and p < 0.0001, (unpaired *t*-test). Values presented as mean ± SEM.

In order to prove that mitochondrial function is crucial for adipogenesis, we treated cells with mitochondrial inhibitors under normoxic conditions and assessed the capacity for differentiation. We treated 3T3-L1 cells under normoxic conditions with a non-toxic concentration (20 nM) of mitochondrial complex *III* inhibitor, antimycin A (AA), for 8 days. A notable decrease in adipogenesis could be seen by the Oil Red O staining test (Fig. 4A). Furthermore, a reduction in movement of the mitochondrial network was observed upon live-cell imaging (Fig. 4B- video available in online version). As mitochondrial movement is dependent on tubulin microtubules, we tested if blocking microtubule polymerization and depolymerization cycles affects mitochondrial movement and henceforth contributes to a reduction of adipogenesis. For this, we treated cells with nocodazole. To avoid interfering with mitotic clonal expansion that occurs during the initial two days after placing cells in the differentiation cocktail, we only treated cells after this event had taken place. As expected, after 8 days, adipogenesis was diminished (Fig. 4C). In agreement with our findings, Woods et al. [37], demonstrated that microtubules are essential for mitochondrial fission and fusion and the disruption of its dynamics is linked with decreased metabolic function, increased ROS production and impairment of ATP generation. Thus, these two different approaches triggering mitochondrial dysfunction could be one of the reasons for the failure of the adipogenic program.

### 3.6 Impairment of the adipogenic potential in an *in vivo SOD1 KO* model

**Fig. 5:**
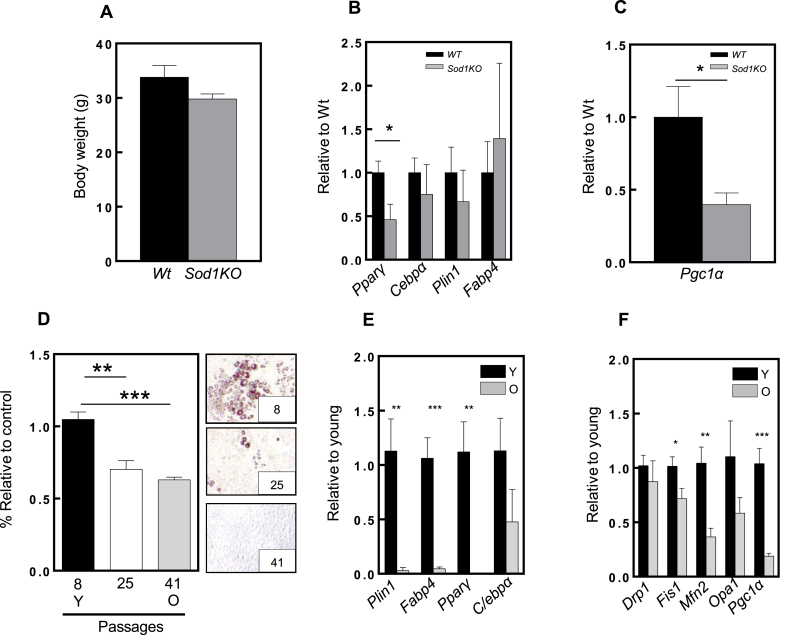
**Adipogenesis is impaired in a mouse model of oxidative stress and accelerated aging.** **(A)** Body weight in grams of Wild type (WT) (black column) and CuZnSOD1KO (SOD1KO) (grey column). **(B)** and **(C)** mRNA levels of adipogenesis and mitobiogenesis markers, respectively, in epididymal white adipose tissue from 8 month-old WT and CuZnSOD1 KO mice. For each qPCR, 4 mice from each group were used. **(D)** Oil Red O staining quantification in different cell passages. The right panels represent the adipogenesis state of each cell passage. **(E)** and **(F)** Adipogenesis and mitodynamics markers in young and old cell passages, respectively (n = 3). Statistical significance was given as follows *p < 0.05, (unpaired *t*-test). Values presented as mean ± SEM.

**Fig. 6:**
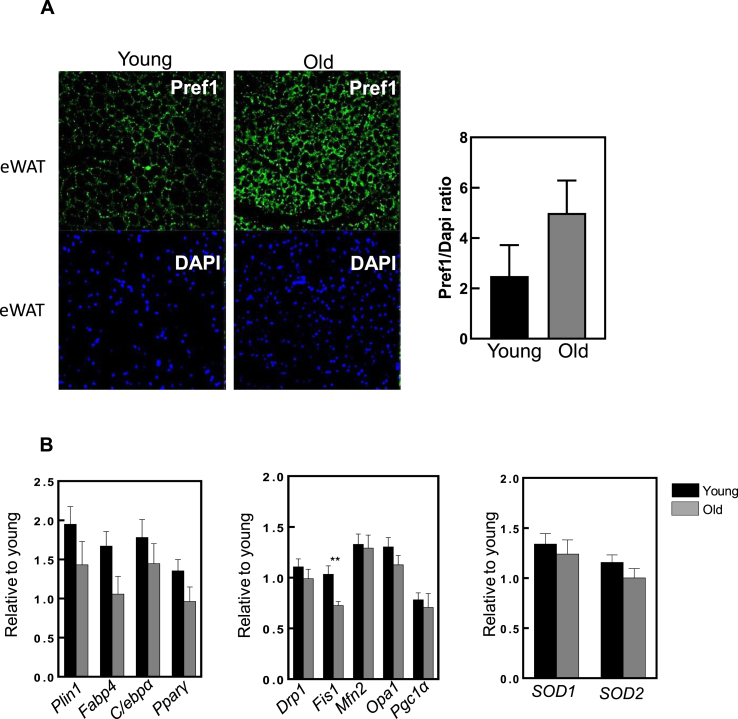
**Adipogenesis in eWAT is impaired in old mice.** **(A)** The upper left panel (young eWAT) shows Pref1 antibody staining when compared with the right (old WAT). The lower panels represent the respective nuclei staining (DAPI). On the right, a representative graphic is observed (n = 3). **(B)** Adipogenesis (left), mitodynamics (middle) and antioxidant enzymes (right) markers in young and old mice eWAT. 7–8 animals were used in each condition. Statistical significance was given as follows **p < 0.01, (unpaired *t*-test). Values presented as mean ± SEM.

**Fig. 7:**
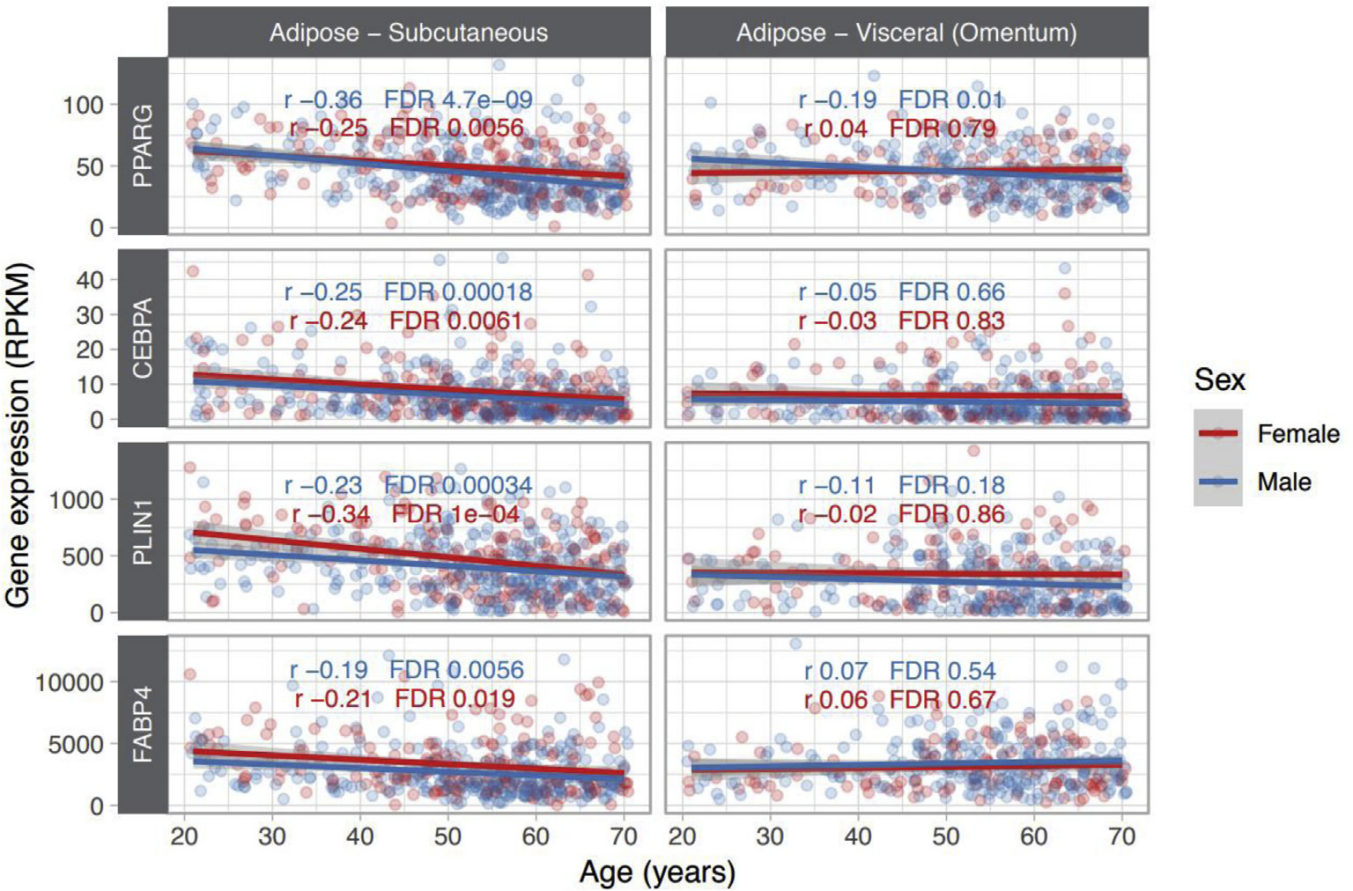
**Crucial adipogenesis genes are downregulated in adipose tissues during human aging.** Plots represent the expression of key genes (*PPARG, CEBPA, PLIN4 and FABP4*) for adipogenesis progression, using GTEx dataset from human subcutaneous (sWAT) and visceral white adipose tissue (vWAT).

We further evaluated the eWAT in an *in vivo* oxidative stress model - mice lacking the Cu/Zn-superoxide enzyme (*Sod1 KO*). These mice were shown to display oxidative stress and accelerated aging. The body weight of these animals was slightly reduced when compared with *Wt* animals, although this did not reach statistical significance (Fig. 5A), as previously shown [24]. In addition, adipogenic genes had a lower expression in *Sod1 KO* mice (Fig. 5B). Although there was increased high variability in *Fabp4* levels, all the other genes related to adipogenesis exhibited a trend for decreased expression, especially the master regulator of adipogenesis, *Pparγ*, and the mitochondrial biogenesis transcription factor, *Pgc1α* (Fig. 5C). This agrees with our *in vitro* model demonstrating the role of oxidative stress in the impairment of adipogenesis. Chronic oxidative stress and dysregulation of gene expression are important features of aged tissues. To investigate whether oxidative stress and aging shared the same pattern in gene expression, we continuously passaged 3T3-L1 preadipocytes (41 passages). Aged cells showed impaired adipogenesis, as observed by Oil Red O quantification (Fig. 5D), in which lipid droplets were reduced in the higher passages. This coincided with a significant downregulation in the expression of genes related to adipogenesis and mitodynamics (Fig. 5E and F, respectively). Similar features were observed *in vivo*, between the eWAT of young and old mice, despite not reaching statistical significance (Fig. 6). Furthermore, the preadipocyte factor 1 (Pref1), a marker of preadipocytes known to inhibit adipogenesis [38], was found higher in old eWAT tissue, yet not significant, suggesting lower adipogenesis during aging could lead to a higher accumulation of precursor cells, preadipocytes (Fig. 6A). In addition, adipogenic and mitodynamic genes previously assessed in cells, showed a similar trend in old tissues (Fig. 6B). It is important to mention that in tissues, the gene expression signals might be diluted due to the presence of other cell types. Nevertheless, these results agree with the genotype-tissue expression (GTEx) dataset [26], wherein adipogenic markers in sWAT such as PPARG, CEBPA, PLIN1 and FABP4, were progressively downregulated during aging in humans (Fig. 7).

## 4 Discussion

Adipogenesis is a physiological process wherein preadipocytes undergo growth arrest, differentiate and accumulate lipids as mature adipocytes, preventing ectopic lipid deposition in peripheral tissues. However, when impaired it can lead to severe consequences such as obesity and insulin resistance. The aging process seems to be responsible for the functional decline of many tissues, including the WAT and therefore its renewal capacity. Oxidative stress is considered to play an important role in the aging process. Mitochondria are crucial for cell energy supply, as they are the main source of ATP, generated via the energy released from oxidation of nutrients. Adipogenesis is a tightly controlled process involving many proteins; among them is *Pparγ*, known as the master regulator. The expression of *Pparγ* is induced in a positive feedback loop by *Cebpα* [39]. These two important transcription factors are responsible for the activation of adipose-specific gene expression enhancers and thus maintain the adipogenesis transcriptional program [40]. Together, they are involved in cellular processes such as proliferation and differentiation [15]. Mitochondria are considered to be, at least in some tissues, the major site for ROS production, which can actually be important for the initiation of *Pparγ* transcriptional machinery by means of redox signaling [15,41]. However, when the physiological levels of ROS increase above a certain threshold, tissues face oxidative stress [42]. Here, we demonstrate that adipogenesis is impaired by oxidative stress and aging, and that this is mediated by mitochondrial dysfunction. Interestingly, we also show that these conditions can be reversed by pharmacological approaches such as treating cells under stress with PPARγ agonists. These observations shed light on chronic hyperoxia-related oxidative stress models of inhibiting adipogenesis, demonstrated by the inability of preadipocytes to differentiate into mature adipocytes (Fig. 1A). Mechanistically, this was shown by the decreased mRNA levels of *Cebpα* and *Pparγ*. Moreover, other adipogenic markers such as *Plin1* and *Fabp4* were lowly expressed in cells exposed to hyperoxia, demonstrated by the absence of lipid droplets.

As expected, in cells exposed to hyperoxia, we observed higher levels of ROS and carbonylated proteins (Fig. 1D and E).

As an irreversible non-enzymatic modification, carbonylation is a marker of severe oxidation of proteins and therefore a reliable marker of oxidative stress. Metabolic dysfunction represented by type 2 diabetes is characterized by elevated ROS levels and oxidative stress [5]. Surprisingly, the amount of carbonylated proteins was decreased when the cells were exposed to a recovery period of eight more days under normoxia conditions, after being exposed for eight days to hyperoxia (Supplemental Fig. 2B). This may be explained by protein turnover through the proteasome when normoxia is restored [43,44] and by the dilution effect of cellular division. Interestingly, under these conditions, adipogenesis was also recovered (Supplemental Fig. 2A).

As already mentioned above, mitochondrial dysfunction can lead to oxidative stress, due to increased ROS production. In our model, this appears to follow a similar mechanism to oxidative phosphorylation complexes; specifically, we observed a downregulation of mitochondrial complex *I* and *II* (Fig. 2A), which probably has an impact on maximal but not basal respiration. Consistent with this observation, there was a close to significant (p = 0.058) decrease in ATP content (Fig. 2C), suggesting altered ATP consumption by the oxidative stressed cells, in which ATP production was not changed (Fig. 2D). A decline in mitochondrial DNA copy number was also observed (Fig. 2B). This finding is expected, since it is known that mitochondrial DNA is abundant during preadipocyte differentiation, and in our oxidative stress model the differentiation process was impaired [15]. Interestingly, cells under hyperoxia showed a lower maximal respiration capacity, presumably as a consequence of the reduction in complex *I* and *II* levels.

After demonstrating how oxidative stress can disrupt adipogenesis, we tested whether a recovery in adipogenesis would be achievable using the antidiabetic drug, rosiglitazone. This drug is an agonist of *Pparγ*, which plays a key role in adipogenesis, as supported by several reports showing its beneficial effects in different diseases with impaired mitochondria [35,45,46]. This was also true in our hyperoxia treated cells, where we could show that R counteracts the negative effects of oxidative stress. In fact, rosiglitazone treatment of cells exposed to hyperoxia could recover adipogenesis (Fig. 3A). This effect could also be a consequence of enhanced mitochondrial biogenesis, seen by the increase of *PGC1α* (Fig. 3A). This was also evidenced by an upregulation of mitochondrial fusion and fission machinery, observed by live-cell imaging (Supplemental Fig. 4A, B and C, respectively), reflecting higher mitochondrial dynamic. Rosiglitazone also rescued mRNA levels of adipogenic markers and mitochondrial fusion-fission machinery (Fig. 3B and E, respectively). Since rosiglitazone is an agonist of *Pparγ*, it seems to directly activate transcription and thus the metabolic program during adipogenesis. Increased fission machinery could also explain the lower amount of carbonylated proteins (Fig. 3C), since fission dilutes the accumulation of DNA mutations and oxidized proteins [47]. However despite this, the difference in levels of oxidative phosphorylation complexes between control and oxidative stressed cells did not reach statistical significance – rosiglitazone seemed to restore complex *I*, *II* and *IV* protein levels. Regarding mitochondrial respiration, we were able to show differences between control and oxidative stressed cells. We observed that in addition to an increase in maximal respiration, basal respiration was also increased. To verify the extent by which mitochondrial function is crucial for adipogenesis, we also tested for cells under normoxia treated with antimycin A, an inhibitor of mitochondria complex *III* (cytochrome c oxidoreductase), resulting in ROS production [48]. After treatment, we showed the same tendency for lower adipogenesis capacity as seen in hyperoxia-treated cells, confirming that mitochondria are crucial for adipogenesis (Supplemental Fig. 4A and B). Another important factor for mitochondrial function is motility. Mitochondria are dynamic organelles that move along microtubules inside the cell, a process which is crucial to their function. Under hyperoxia conditions, we showed impairment in mitochondrial mobility and function. Adding to this, the reduction of mitochondrial dynamic by the microtubule inhibitor nocodazole achieved a similar level of adipogenesis reduction (Fig. 4C). We observed that adipogenesis declined after 8 days of nocodazole treatment, reinforcing the importance of mitochondria motility and dynamics.

As we demonstrated that “unhealthy” mitochondria lead to impaired adipogenesis in 3T3-L1 cells, either by hyperoxia or with use of mitochondrial function/dynamics inhibitors, we examined whether our findings are transferable to an *in vivo* situation. We took advantage of an *in vivo* mouse model lacking an important mitochondrial and cytoplasmic antioxidant enzyme, superoxide dismutase 1 (SOD1), which is prone to increased levels of oxidative stress. Indeed, Vasilaki et al. [49] have shown that these mice display higher levels of protein carbonyls and 3-nitrotyrosine, two markers of oxidative damage. We collected the eWAT from these mice. As we analyzed the whole tissue and not just individual preadipocytes, we focused mainly on the important adipogenic transcription factors, *Cebpα* and *Pparγ* (Fig. 5B), and the master regulator of mitochondrial biogenesis, *Pgc1α* (Fig. 5C). Probing for these specific genes allowed us to assess a type of “adipogenic potential”, since we could not examine the cells of interest individually. These markers tended to be decreased in mice lacking superoxide dismutase, supporting our studies *in vitro*.

These results suggest that oxidative stress plays a detrimental role in adipogenesis.

Oxidative stress accumulates with aging and therefore one would expect decreased adipogenesis in aged eWAT. In fact, when staining eWAT tissue for Pref1, a preadipocyte marker, we observed a trend for increased Pref1 in old eWAT, suggesting that the old mice may have more preadipocytes, and thus less adipogenic capacity compared to young animals (Fig. 6A). With regard to adipogenesis, mitodynamics and antioxidant markers, these were slightly reduced in the old tissue, suggesting an adipogenic impairment during aging (Fig. 6B). This appears to have the same effect in human subjects. Taking advantage of the GTEx dataset, we found that the expression of 4 analyzed genes (PPRAG, CEBPA, PLIN4 and FABP4) was significantly reduced with age. This effect was more pronounced in subcutaneous fat tissue than in visceral fat. The human data helps to elucidate why in our mouse epididymal adipose tissue we observe trends, but not significant changes, during aging. Our data suggest that different WAT should be carefully and independently studied, as it is known that subcutaneous adipose tissue has higher basal levels of adipogenic genes, such as *Pparγ* and *Cebpα* [50], pointing to different regulation and metabolism in these types of adipose tissue.

In conclusion, our results emphasize that functional and dynamic mitochondria are crucial for the adipocyte differentiation process. This was demonstrated not only *in vitro,* but also in the analyzed mice models and in humans. However, despite being used for diabetes type II treatment, it remains unclear whether rosiglitazone could restore the adipogenesis process during aging. Additional studies are needed to demonstrate its efficacy in the treatment of obesity and aging.

## Funding

This work was funded by the German Center for Diabetes Research (DZD), the *Gesundheitscampus* Brandenburg and the German Center of Cardiovascular Research (DZHK), partner site Berlin. VNG is funded by grants from the National Institute of Health (NIH), Bethesda, USA.

## Declaration of competing interest

The authors declare no conflict of interest.

## Appendix A Supplementary data

Figures.pptx [8-11] **Supplemental Fig.1. Volcano plot indicates stress response in hyperoxia treated cells.** The square on the plot depicts upregulated protein on the hyperoxia treated cells. Using *String* for enrichment analyses annotated pathways for stress and aging were found. **Supplemental.Fig. 2 Hyperoxia leads to mild and non-cytotoxic oxidative stress after 8 days.** In both graphics, black dashed line represents OS while grey box represents 8 days recover (OS+Rec) at normoxia (21%O2) after 8 days in hyperoxia (40%O2). All data are relative to control. **(A)** Oil Red O staining quantification (n = 3). **(B)** Quantification of protein carbonylation in comparison with normoxia cells. (n = 3). Statistical significance was given as follows *p < 0.05, (unpaired *t*-test). Values presented as mean ± SEM. **Supplemental.Fig. 3 Hyperoxia slightly reduces mitodynamics and mitobiogenesis. (A)** represents the densiometric quantification of mitochondrial fusion marker mitofusin (Mfn2) and **(B)** and **(C)** represent the densiometric quantification of mitochondrial fission markers, Fis1 and DRP1, respectively. **(D)** represents the densiometric quantification of mitochondrial biogenesis marker, PGC1α. In the four graphics, the black columns represent normoxia cells (C) and the grey columns represent oxidative stress/hyperoxia (OS) cells. All data is relative to control (n = 3). **(E)** The panels represent live cell imaging of mitochondria, where these were stained with Mitotracker. The left panel represents the mitochondrial movement in control cells (normoxia), while the right panel represents the mitochondrial movement in oxidative stressed cells. The panels show one representative video of each condition, from three independent experiments (n = 3). These videos can be seen in the online version. **(F)** mRNA levels of mitodynamic (*Drp1*, *Fis1*, *Mfn2* and *Opa1*) and mitobiogenesis (*PGC1α*) genes in normoxia (black columns) and hyperoxia (grey columns) cells. Samples are relative to control (n = 3). Values presented as mean ± SEM. **Supplemental.Fig. 4 Rosiglitazone improves mitochondrial network. (A)** and **(B)** represent the densiometric quantification of mitochondrial fusion (Mfn2) and fission (DRP1) markers, respectively, in OS and OS+Rosi treated cells. Samples are relative to control and for each protein three independent experiments were done (n = 3). Statistical significance was given as follows *p < 0.05 and **p < 0.01 (unpaired *t*-test). Values presented as mean ± SEM. **(C)** The panel represents live cell imaging after 8 days of mitochondria treatment with rosiglitazone for 5 days under hyperoxia conditions, where these were stained with Mitotracker. The panel shows one representative video of this condition (n = 3). Figures.pptx [8-11]
